# Whale Müller glia create a permissive environment supporting retinal ganglion cell survival and neurite outgrowth

**DOI:** 10.1016/j.isci.2026.117637

**Published:** 2026-09-25

**Authors:** Xandra Pereiro, Noelia Ruzafa, Laura Prieto-López, Marcel Blindert, Stefanie M. Hauck, Félix Elortza, Mikel Azkargorta, Elena Vecino

**Affiliations:** 1 Experimental Ophthalmo-Biology Group, Department of Cell Biology and Histology, University of the Basque Country UPV/EHU, Leioa, Spain; 2 Begiker-Ophthalmology Research Group, BioBizkaia Health Research Institute, Cruces Hospital, Barakaldo, Spain; 3 Metabolomics and Proteomics Core, Helmholtz Centre Munich, German Research Center for Environmental Health, Neuherberg, Germany; 4 Proteomics Platform, CIC BioGUNE, Basque Research and Technology Alliance (BRTA), CIBERehdProteoRed-ISCIII, Bizkaia Science and Technology Park, Derio, Spain

**Keywords:** whale, Müller glia, retinal ganglion cells, neurite outgrowth, neuron survival

## Abstract

The adult central nervous system has limited regenerative capacity, with retinal ganglion cells (RGCs) particularly refractory to repair. The extracellular glial environment can critically influence neuronal survival and regenerative responses. Here, using cross-species co-culture, conditioned-medium approaches, quantitative proteomics, and functional perturbation, we characterize a Müller glial phenotype from large baleen whales that supports RGC survival and neurite extension. Whale Müller glia enhanced neuronal viability and neurite elongation compared with pig and rat Müller glia. Proteomic analyses revealed species-dependent differences in conditioned-medium composition, including greater abundance of mesencephalic astrocyte-derived neurotrophic factor (MANF) in whale conditioned medium and detection of the axon-repulsive protein fibronectin leucine-rich transmembrane protein 2 (FLRT2) in pig conditioned medium. FLRT2 suppression combined with MANF enhanced neurite outgrowth in a subset of cells within RGC-enriched cultures only in the presence of Müller glia. These findings identify glia-dependent molecular signals associated with a permissive retinal environment supporting neuronal survival and neurite outgrowth.

## Highlights

• Whale Müller glia enhance retinal ganglion cell survival
• Whale Müller glia promote retinal ganglion cell neurite outgrowth over rat and pig Müller glia
• Species-specific Müller glial secretomes differ in trophic and inhibitory signals
• Müller glia actively shape the extracellular environment regulating neurite outgrowth

## Graphical abstract

**Figure:**
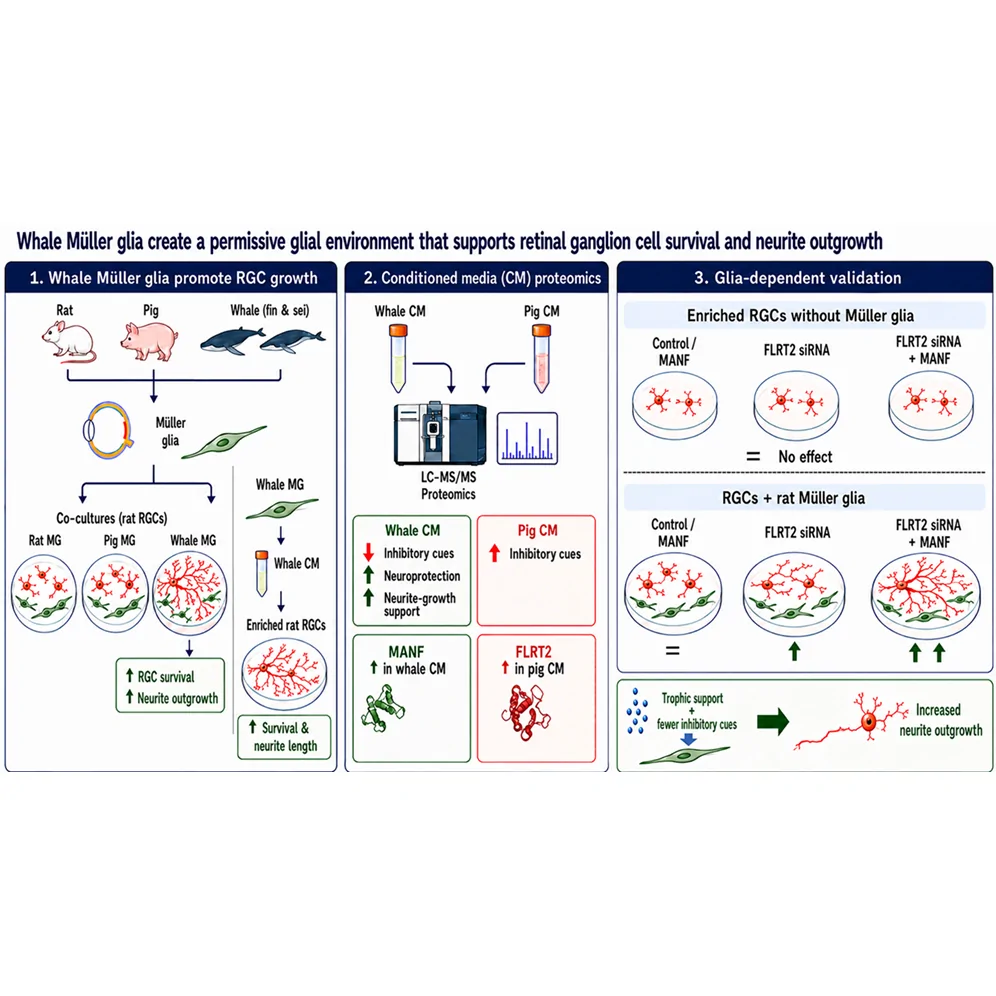

## Teaser

Neuroscience; Sensory neuroscience; Cell biology

## Introduction

The failure of the central nervous system (CNS) to regenerate after injury is attributed both to diminished intrinsic growth capacity of adult neurons (cell-intrinsic barriers) and to lack of physical support and inhibitory signals present in the extracellular environment (cell-extrinsic barriers).^1^^,^^2^^,^^3^ Many widely used strategies to induce long-distance axon regeneration focus on overcoming cell-intrinsic barriers, often through the upregulation of dormant signaling pathways that were active during neuronal development; however, complementary approaches also target cell-extrinsic constraints by modulating the injury milieu and augmenting growth-permissive substrates.

Despite these advances, regenerating retinal ganglion cell (RGC) axons frequently stall and fail to sustain long-distance extension,^4^ and when axons do extend they can also show targeting and guidance defects, frequently halting at the optic chiasm or growing aberrantly. Even when RGCs axons successfully grew beyond the chiasm, they primarily projected to bilateral suprachiasmatic nuclei (SCN), with only a limited number reaching distal visual centers such as the superior colliculus (SC) or lateral geniculate nucleus (LGN).^5^^,^^6^

RGCs provide a well-established and experimentally robust model for studying regeneration due to their well-defined functions, accessibility, anatomical localization, and established injury models. Following injury, these axons traverse regions containing multiple injury responsive resident glial cell types, including astrocytes, microglia, Müller glia in the retina, and oligodendrocytes in the optic nerve, together with infiltrating immune cells such as macrophages and neutrophils. Failure of regeneration is primarily due to both external inhibitory signals and intrinsic growth constraints in mature neurons.^1^^,^^2^^,^^7^^,^^8^^,^^9^ CNS regeneration is inhibited by three main classes of extracellular factors: axon guidance molecules,^9^^,^^10^^,^^11^ myelin-associated inhibitors,^6^^,^^12^ and chondroitin sulfate proteoglycans.^13^^,^^14^ Initial approaches aimed to neutralize these inhibitory factors to promote axon regeneration,^15^ but more recent studies have focused on enhancing the intrinsic regenerative capacity of injured neurons.^16^^,^^17^

Müller glial cells play a pivotal role in retinal homeostasis and have been implicated in both neuroprotection and regenerative responses following injury. Furthermore, Müller glia influence RGCs neurite outgrowth by secreting neurotrophic factors and by triggering specific intracellular cascades.^18^^,^^19^^,^^20^^,^^21^ In several animal, *in vitro*, and co-culture studies, activation of the Wnt/β-catenin pathway by factors such as Norrin increased expression of mediators like β-catenin, leukemia inhibitory factor (LIF), and brain-derived neurotrophic factor (BDNF), are involved in a series of events reported to enhance neurite length and branching.^22^ BDNF-TrkB signaling, particularly after optic nerve injury, was shown to upregulate basic fibroblast growth factor (FGFb), a response linked to early RGC survival and potential neurite extension.^23^ In parallel, Müller glia purinergic signaling—primarily via ATP acting on P2Y6 receptors—was reported to be directly associated with increased neurite outgrowth.^24^ Additional studies documented that STAT3 activation, along with participation of MAPK and NF-κB pathways, contributes to neuroprotective and axon-growth responses.^25^ These convergent signaling mechanisms underscore the role of Müller glia as active modulators of neurite development in RGCs.^26^ However, despite these promising findings, functional recovery remains limited, and neurodegeneration continues to progress. Therefore, additional strategies that not only promote axon growth but also ensure neuronal survival are urgently needed for functional restoration.

In the present study, we investigated a serendipitous observation: Müller glial cells derived from whales promoted RGC neurite outgrowth *in vitro*. Given this unexpected result, we repeated the experiments using Müller glia from two different whale specimens, confirming the reproducibility of the observed effect. These findings indicate that whale Müller glia provide a supportive environment for neuronal survival and neurite outgrowth and offer new insights into glia-mediated regulation of neuronal growth. We subsequently explored the molecular and cellular mechanisms potentially contributing to this effect and considered their relevance to neuronal repair in the retina and broader CNS.

In order to further validate these findings, we conducted RGC co-culture experiments with whale Müller glial cells and confirmed the results using conditioned media (CM) from whale Müller glia. Additionally, we compared these effects with Müller glial cells derived from pig and rats to assess species-specific differences. To identify the molecular components involved in the enhanced neurite elongation paradigm, we performed a comparative proteomic analysis of the CM from both whale and pig Müller glia, in order to pinpoint the key molecule(s) responsible for its enhanced regenerative capability.

## Results

### Survival and neurite outgrowth of rat RGCs in co-culture with whale Müller glia

To assess the effects of whale Müller glia on rat RGCs, we established a co-culture system and analyzed RGC survival and neurite outgrowth (Figure 1A).

**Figure 1:**
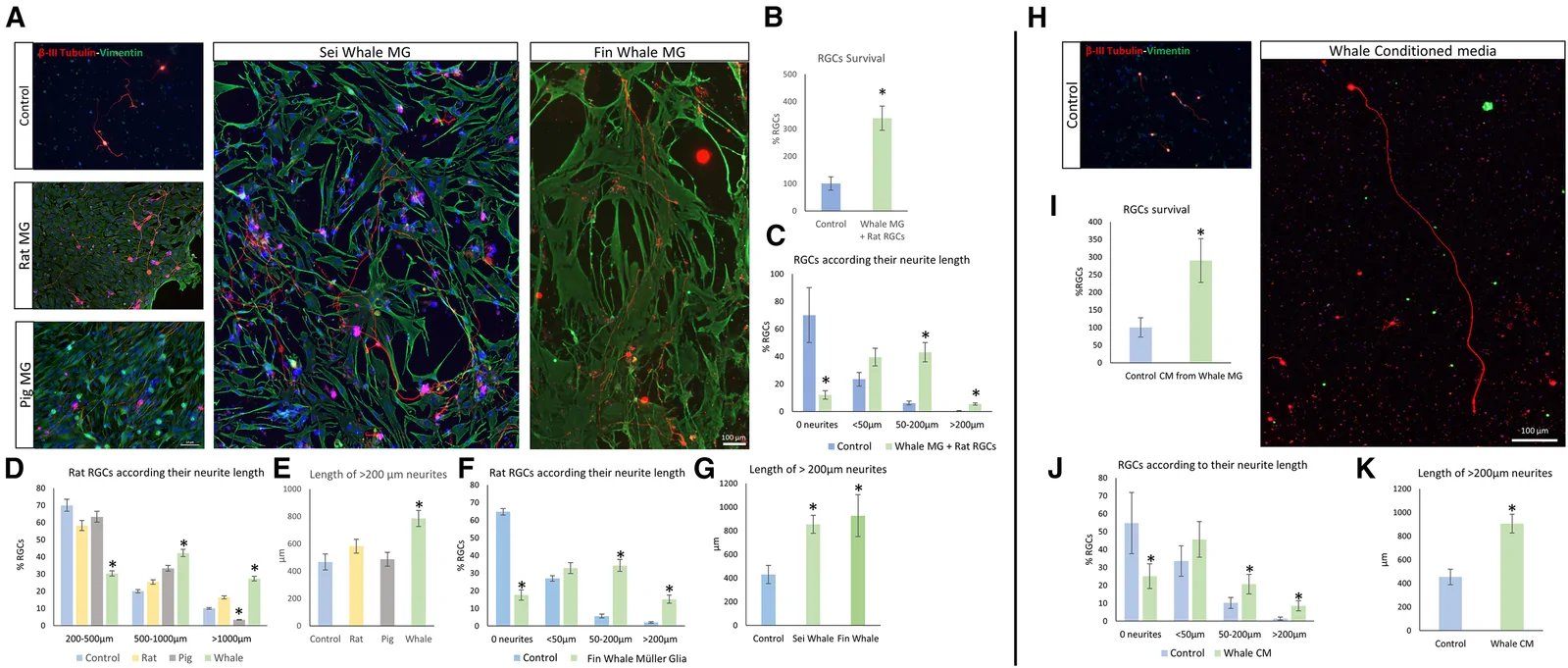
Species-dependent effects of Müller glia co-culture and whale Müller conditioned media on rat RGC survival and neurite outgrowth (A) Images of rat RGCs from control (RGC-enriched cultures) and in co-culture with rat, pig, and whale Müller glia at 6 days *in vitro* (DIV), RGCs (red) labeled with an antibody against β-III-tubulin and Müller glia (green) labeled with an antibody against vimentin, nuclei (blue) stained with DAPI. (B) Analysis of the survival of rat RGCs in co-culture with whale Müller glia. Survival was expressed as a percentage relative to the control, RGC-enriched condition (100%). Whale Müller glia significantly increase the survival of RGCs. (C) Analysis of the percentage of RGCs with, no neurites, short (<50 μm), medium (50–200 μm), and long (>200 μm) neurites from control and in co-culture with whale Müller glia. (D) Percentage of rat RGCs with 200–500, 500–1,000, and >1,000 μm neurites under control conditions and in co-culture with rat, pig, and whale Müller glia. Whale Müller glia significantly enhance neurite outgrowth in rat RGCs. (E) Quantification of the neurites (μm) of RGCs with extended neurites (>200 μm) under control conditions and in co-culture with rat, pig, and whale Müller glia. Whale Müller glia significantly increase the length of RGCs with >200 μm neurites. (F) Percentage of rat RGCs with no neurites, short (<50 μm), medium (50–200 μm), and long (>200 μm) in culture with fin whale Müller glia. (G) Quantification of the neurites (μm) of RGCs with extended neurites (>200 μm) under control conditions (RGC-enriched culture) and with fin and sei whale Müller glia. Notice that both Müller glia from fin and sei whale increase the neurite outgrowth of rat RGCs compared to control condition (RGC cultures maintained without Müller glia). (H) Images of rat RGC-enriched cultures from control (without whale CM) and with whale Müller glia CM at 6 DIV, RGCs (red) labeled with an antibody against β-III-tubulin and Müller glia (green) labeled with an antibody against vimentin, nuclei (blue) stained with DAPI. (I) Analysis of the survival of rat RGCs with whale Müller glia CM. Survival was expressed as a percentage relative to the control condition (100%). CM from whale Müller glia significantly increase the survival of RGCs. (J) Analysis of the percentage of RGCs with, no neurites, short (<50 μm), medium (50–200 μm), and long (>200 μm) neurites from control and with whale Müller glia CM. (K) Quantification of the neurites (μm) of RGCs with extended neurites (>200 μm) under control conditions and with whale Müller glia CM. Scale bars: 100 μm. ∗ indicates *p* < 0.05; statistical analyses were performed using (B, E, H, and J) Mann-Whitney U test analysis and (C–D, F, and I) Kruskal-Wallis nonparametric test. Data represented by mean ± SEM. Data represent 3 independent experiments (3 replicates from each).

Quantification of RGC survival showed a significant increase in the presence of whale Müller glia (339.10% ± 44.45%) compared to highly enriched RGCs cultures as control condition (100% ± 24.40%) (Figure 1B). Additionally, we evaluated neurite outgrowth by classifying RGCs based on neurite length into four categories: no neurites, short (<50 μm), medium (50–200 μm), and long (>200 μm).

### Comparative analysis of neurite outgrowth in rat RGCs co-cultured with rat, pig, and whale Müller glia

Next, to compare the effects of Müller glia from different species on rat RGC neurite outgrowth, we co-cultured RGCs with rat, pig, or whale Müller glia and analyzed neurite extension. Categorization of RGCs based on neurite length (200–500, 500–1,000, and >1,000 μm), showed a higher percentage of RGCs with longer neurites (>1,000 μm) in co-culture with whale Müller glia (27.39% ± 1.36%) compared to rat (16.41% ± 0.82%) and pig (3.33% ± 0.16%) Müller glia (Figure 1D). As a control, RGCs were cultured in the absence of Müller glia.

Quantification of neurite length in RGCs with extended neurites (>200 μm) revealed that whale Müller glia significantly increased RGCs neurite length (784.72 ± 59.54 μm) compared to rat (581.77 ± 51.68 μm) and pig (486.53 ± 50.66 μm) Müller glia (Figure 1E). The control consisted of RGC cultures without Müller glia. These findings suggest that whale Müller glia provide a neuroprotective and growth-promoting environment for rat RGCs compared to Müller glia from rat and pig.

To evaluate whether the immortalization process and cellular aging (reflected by advanced passages) affect the neuroprotective and growth-promoting properties of whale Müller glia, we performed a comparative analysis of rat RGC neurite outgrowth in co-cultures with immortalized and primary whale Müller glia at passages 4 and 7 (Figure S1). Quantification of RGCs with neurites revealed that primary whale Müller glia significantly increased the percentage of RGCs bearing 200–500 (95.00% ±8.36%), 500–1,000 (197.91% ± 52.40%), and >1,000 μm neurites (71.25% ± 2.26%) compared to control cultures (without Müller glia) (33.33% ± 20.99%, 41,66% ± 15,95%, and 25.00% ± 1.99%, respectively), and notably, this neurotrophic effect was enhanced at higher passages (290.27% ± 48.48%, 250.69% ± 12.12%, and 105.55% ± 48.48%, respectively). In contrast, immortalized Müller glia (W21M) lost the ability to promote longer neurites (>1,000 μm), showing no significant improvement over control conditions (24.66% ± 2.31%). Additionally, analysis of the absolute neurite length in RGCs with neurites longer than 200 μm demonstrated that only primary whale Müller glia significantly extended neurite length (1184.46 ± 140.05 μm at passage 4 and 1153.82 ± 104.17 μm at passage 7) compared to both control (749.29 ± 209.60 μm) and immortalized (992.59 ± 191.56 μm) Müller glia. These findings indicate that while primary whale Müller glia retain their capacity to support RGC neurite outgrowth, the immortalization process leads to a loss of this elongation-promoting property.

### Neurite outgrowth of rat RGCs in co-culture with fin whale and sei whale Müller glia

To evaluate the effect of Müller glia from different whale species on rat RGC neurite outgrowth, we co-cultured RGCs with Müller glia from fin whale (Figure 1A) and assessed neurite extension, comparing it with sei whale Müller glia (used in the previous experiments). Quantification of neurite length categorized RGCs into four groups: no neurites, short (<50 μm), medium (50–200 μm), and long (>200 μm) showing that co-culture with Müller glia from fin whale also significantly increased the proportion of RGCs with medium (41.86% ± 3.39%) and long neurites (15.21% ± 2.27%) compared to control (without Müller glia) conditions (5.65% ± 1.08% and 2.03% ± 0.45%, respectively) (Figure 1F).

Further analysis of neurite extension in RGCs with long neurites (>200 μm) demonstrated that Müller glia from both fin (927.35 ± 177.16 μm) and sei whale Müller glia (854.14 ± 76.24 μm) significantly enhanced neurite outgrowth compared to control conditions (429.80 ± 77.37 μm) (Figure 1G). These findings suggest that Müller glia from both whale species provide a growth-promoting environment for rat RGCs.

### Survival and neurite outgrowth of rat RGCs in co-culture with whale Müller glia CM

To evaluate the effects of whale Müller glia CM on rat RGCs, we analyzed both cell survival and neurite outgrowth (Figure 1H).

Quantification of RGC survival revealed a significant increase in the presence of whale Müller glia CM (289.85% ± 62.27%) compared to control (without whale CM) condition (100% ± 27.55%) (Figure 1I). To assess neurite outgrowth, RGCs were categorized based on neurite length as follows: no neurites, short (<50 μm), medium (50–200 μm), and long (>200 μm). The proportion of RGCs with extended neurites (8.57% ± 2.78%) was significantly higher when cultured with whale Müller glia CM compared to control (1.45% ± 0.84%) (Figure 1J).

Further analysis demonstrated that RGCs with long neurites (>200 μm) exhibited significantly increased neurite length in the presence of whale Müller glia CM (904.91 ± 79.14 μm) compared to control condition (454.27 ± 65.85 μm) (Figure 1K). These findings indicate that soluble factors secreted by whale Müller glia contribute to RGC survival and neurite growth.

### Proteomic analysis of CM from whale and pig Müller glia

To obtain a functional overview of the secreted protein profiles potentially associated with neurite elongation, we performed a proteomic analysis on the CM collected from Müller glia of the sei whale and domestic pig. The identified proteins were functionally annotated according to their reported biological roles and classified into categories relevant to neuronal growth, including neuroprotection, positive regulation of neurite outgrowth, and negative regulation of neurite outgrowth, among others. The purpose of this analysis was to provide biological context for the observed functional differences between conditions rather than to perform quantitative comparison or statistical enrichment analysis between species. Accordingly, the reported percentages represent the proportion of identified proteins assigned to each functional category relative to the total number of proteins detected within each species-specific dataset, and these functional annotations should be interpreted as a descriptive representation of the secretome composition in each species.

Proteomic analysis of CM from Müller glia of the sei whale and domestic pig revealed notable differences in the composition of secreted proteins related to neuronal growth and regeneration. In whale Müller glia CM (1,200 proteins identified), proteins associated with neuroprotection (7.4%), and positive regulation of neurite outgrowth (7.1%) were markedly more abundant than in pig Müller glia CM (747 proteins identified), where these categories represented only 3.2% and 3.6%, respectively. Proteins associated with the negative regulation of neurite outgrowth represented a smaller proportion of the proteins identified in whale CM (0.2%) than in pig CM (3.3%), suggesting differences in the representation of potentially inhibitory signals between the two secretomes.

Other functional categories also differed between species: whale CM showed lower proportions of proteins involved in cytoskeleton organization, adhesion, and migration (11.8%) and inflammation (2.5%) compared to pig CM (19.0% and 12.3%, respectively). Proliferation/mitosis-related proteins were slightly more represented in whale CM (6.5%) than in pig CM (4.7%) (Figure 2).

**Figure 2:**
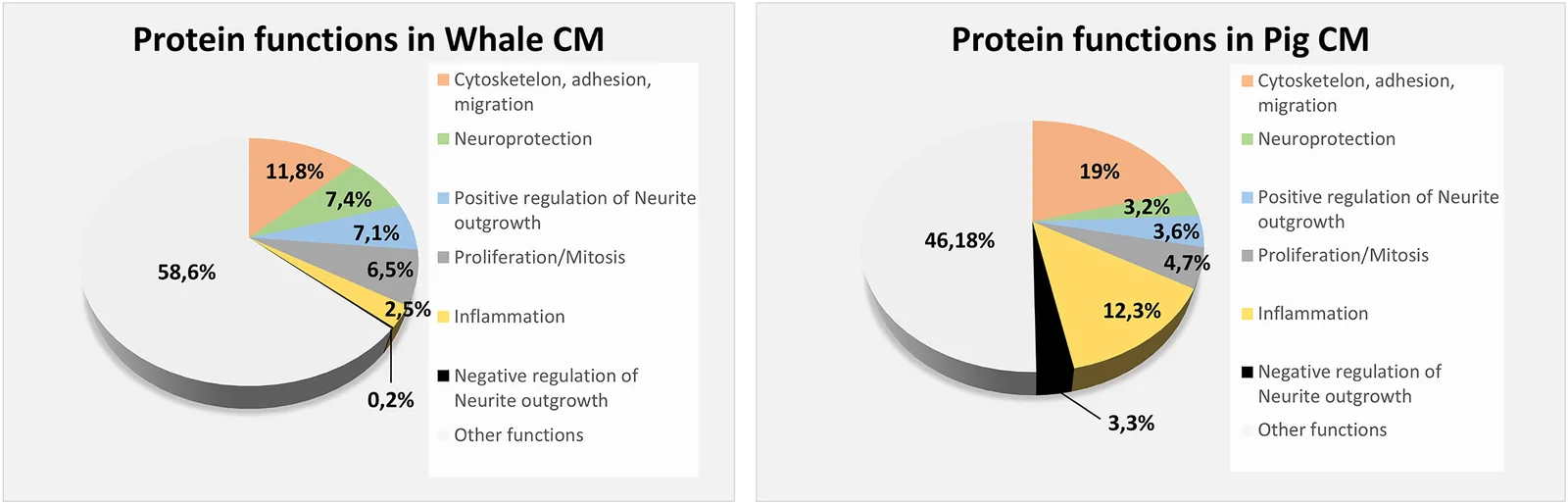
Proteomic analysis of conditioned media from whale and pig Müller glia Functional annotation of proteins identified in the CM obtained from whale (left) and pig (right) Müller glia. Proteins detected in each conditioned medium were assigned to functional categories according to their reported biological functions. Percentages represent the proportion of proteins associated with each category relative to the total number of proteins identified within the corresponding species-specific dataset. This analysis is intended as a descriptive overview of secretome composition.

These comparative findings demonstrate that CM from sei whale Müller glia is enriched in neuroprotective and neurite-promoting proteins and contains fewer proteins inhibiting neurite outgrowth, suggesting that whale Müller glia provide a more neuroprotective and pro-regenerative environment for RGCs compared to pig Müller glia.

These findings suggest that whale Müller glia CM may provide a more neuroprotective and pro-regenerative environment for RGCs compared to pig Müller glia CM.

Although several proteins exclusively present or overrepresented in whale Müller glia CM—such as EPHB3, TBC1D23, DPYSL5, and IGSF8 (Table 1)—have been reported in contexts related to neuronal development, axon guidance, or neuronal survival, their specific contribution to neurite elongation and neuronal survival within the context of Müller glia-mediated effects remains incompletely characterized. Several of these candidates participate in signaling pathways whose biological effects are highly dependent on cellular context, developmental stage, and injury state.

**Table 1:** Proteins identified in the proteomic analysis of whale CM associated with the positive regulation of neurite outgrowth

Positive regulation of neurite outgrowth	Fold (whale/pig) whale FASTA database	Fold (whale/pig) human FASTA database	Fold (whale/pig) pig FASTA database	Mean fold
EPHB3	100.0	100.0	100.0	100.0
TBC1D23	100.0	100.0	100.0	100.0
DPYSL5	100.0	100.0	100.0	100.0
IGSF8	–	100.0	100.0	100.0
GSTP1	100.0	100.0	–	100.0
MOV10	100.0	3.6	3.9	35.8
DRP2	24.6	–	41.9	33.3
MANF	8.6	13.1	14.4	12.0
FLNC	10.9	13.5	5.8	10.1
OGN	6.8	10.5	12.4	9.9
HSPA5	5.2	8.2	9.0	7.5
THBS2	3.5	6.3	6.2	5.3
DCLK1	–	4.6	4.8	4.7
HSP90AB1	3.1	4.7	5.2	4.3
NUDC	2.6	4.2	3.9	3.6
MAP2	2.2	3.4	3.7	3.1
MMP2	2.1	3.3	3.7	3.0
USP9X	–	2.6	3.5	3.0
CNPY2	–	3.1	2.9	3.0
CPNE1	–	2.7	3.2	2.9
GDI1	–	2.8	3.0	2.9
HSP90AA1	2.4	3.8	2.3	2.8
PAFAH1B3	–	2.5	3.0	2.8
QKI	2.1	2.3	2.6	2.3
SMARCC2	–	2.1	2.3	2.2
DYNC1LI2	–	2.1	2.4	2.2
EIF2B2	–	2.1	2.3	2.2
LAMB2	–	2.1	2.2	2.1
KPNB1	–	2.6	3.2	2.9
RACK1	2.2	2.7	3.3	2.8
TGFB2	2.2	3.4	3.8	3.2
JUP	–	3.0	3.2	3.1
PPP2R5D	2.3	3.7	4.1	3.4
PDGFB	–	2.9	3.0	2.9

Proteins were selected based on functional annotation using a fold-change threshold >1.5 (whale/pig) across multiple database searches.

Mesencephalic astrocyte-derived neurotrophic factor (MANF) was particularly noteworthy due to its consistently elevated abundance in whale CM relative to pig CM (mean fold change = 12.0). Combined with its well-established neuroprotective and neurite-promoting properties across multiple neuronal paradigms, MANF was selected as the most suitable candidate for functional validation as a potential mediator of the enhanced neurite outgrowth observed with whale Müller glia CM.

Proteomic analysis of CM from whale and pig Müller glia additionally identified multiple proteins associated with neuroprotective pathways, several of which were highly enriched in whale CM (Table 2). Among them, charged multivesicular body protein 6 (CHMP6), migration and invasion enhancer 1 (MIEN1), glutathione S-transferase P (GSTP1), glutathione peroxidase 4 (GPX4), oxidation resistance 1 (OXR1), and charged multivesicular body protein 4B (CHMP4B) were exclusively detected in whale CM, suggesting a secretome composition potentially associated with increased neuro-supportive capacity.

**Table 2:** Proteins identified in the proteomic analysis of whale CM associated with neuroprotection

Neuroprotection	Fold (whale/pig) whale FASTA database	Fold (whale/pig) human FASTA database	Fold (whale/pig) pig FASTA database	Mean fold
CHMP6	100.0	100.0	100.0	100.0
MIEN1	100.0	100.0	100.0	100.0
GSTP1	100.0	100.0	–	100.0
GPX4	100,.0	100.0	–	100.0
OXR1	100.0	100.0	–	100.0
CHMP4B	100.0	100.0	–	100.0
KRT8	100.0	2.2	8.4	36.9
TXNDC5	23.7	24..3	–	24.0
HSP90B1	15.1	21.8	25.7	20.9
HYOU1	7.7	14.2	14.1	12.0
PRDX4	12.8	15.8	5.9	11.5
KRT18	–	14.4	7.3	10.9
ASNS	6.8	10.7	11.9	9.8
ERO1A	5.2	8.2	9.7	7.7
HSPA5	5.2	8.2	9.0	7.5
MY018A	5.0	7.6	8.8	7.1
HDGF	–	2.0	8.6	5.3
TXNDC12	–	4.5	5.1	4.8
PSMB6	3.1	4.8	5.3	4.4
GCLC	–	4.0	4.4	4.2
MIF	–	4.2	4.1	4,.1
ARL6IP1	2.3	3.6	4.2	3.4
PSMC5	2.2	3.4	3.9	3.1
COPS5	–	2.8	3.2	3.0
PSMC2	–	2.9	3.0	2.9
PDGFB	–	2.9	3.0	2.9
PSME3	–	2.8	2.9	2.8
PRDX6	2.1	2.8	3.5	2.8
TIAL1	–	2.6	2.9	2.7
TPP2	2.1	2.9	3.1	2.7
NPM1	2.2	2.7	3.0	2.6
NME2	–	2.8	2.4	2.6
PSMB4	–	2.5	2.4	2.5
GSR	–	2.1	2.5	2.3
DDB1	–	2.1	2.4	2.2

Proteins were selected based on functional annotation using a fold-change threshold >1.5 (whale/pig) across multiple database searches.

Proteomic analysis of CM from whale and pig Müller glia revealed a subset of proteins associated with the negative regulation of neurite outgrowth (Table 3). Several of these proteins, including collagen type III alpha 1 chain (COL3A1), frizzled-related protein (FRZB), slit guidance ligand 3 (SLIT3), ephrin A1 (EFNA1), and semaphorin 3D (SEMA3D), were exclusively identified in pig CM, suggesting the presence of candidate inhibitory cues within the pig secretome. Among the identified candidates, fibronectin leucine-rich transmembrane protein 2 (FLRT2) was of particular interest due to its well-documented role in axon guidance and neurite repulsion. FLRT2 was exclusively detected in pig CM, with a fold change of 100, indicating its absence or minimal expression in whale CM. This observation identified FLRT2 as a suitable candidate to experimentally test whether differential presence of inhibitory signals contributes to species-dependent differences in neurite extension.

**Table 3:** Proteins identified in the proteomic analysis of pig CM associated with the negative regulation of neurite outgrowth

Negative regulation of neurite outgrowth	Fold (pig/whale)
COL3A1	100.0
FRZB	100.0
SLIT3	100.0
EFNA1	100.0
FLRT2	100.0
ARSB	100.0
SEMA3D	100.0
OLFM1	100.0
CHRD	100.0
MARCKSL1	100.0
TMOD2	24.4
S100B	8.6
EFMP1	7.4
EPS8	5.6
PALLD	5.3
NRP2	5.0
GFAP	4.9
NEXN	4.7
ITM2C	4.6
WNT5A	3.1
CNTN1	2.9
PTPRG	2.5
DPYSL3	2.1

Proteins were selected based on functional annotation using a fold-change threshold >1.5 (pig/whale) across multiple database searches.

Therefore, we prioritized MANF and FLRT2 for functional validation based on three complementary criteria: differential representation in the proteomic datasets, biological plausibility, and functional complementarity. MANF was strongly enriched in whale Müller glia CM, whereas FLRT2 was exclusively detected in pig CM, making them among the most discriminative candidates identified in our proteomic analysis. In addition, both proteins represent biologically distinct and complementary regulatory mechanisms of neurite growth MANF as a well-characterized neurotrophic and pro-regenerative factor, and FLRT2 as a guidance-related inhibitory cue. This combination allowed us to experimentally evaluate whether species-specific secretome composition actively contributes to shaping neurite behavior.

### Effect of the addition of MANF and FLRT2 inhibition on neurite outgrowth in rat RGCs co-cultured with rat Müller glia and in highly enriched RGC cultures

To evaluate the impact of MANF and FLRT2 inhibition on neurite outgrowth, we analyzed rat RGCs co-cultured with rat Müller glia and in RGC-enriched cultures under different experimental conditions.

In co-cultures RGC + Müller glia, quantification of neurite length (>200 μm) revealed that FLRT2 silencing promoted neurite elongation (1101.85 ± 111.70 μm), suggesting that the inhibition of this negative regulator creates a more permissive environment for neurite extension when is compared to control (co-cultures without any treatment) condition (730.81 ± 57.83 μm). Notably, the combined treatment of FLRT2 small interfering RNA (siRNA) + MANF resulted in a synergistic effect, further increasing neurite length (1359.73 ± 180.88 μm). However, treatment with MANF alone did not result in any increase in the length of the longest neurites compared to the control condition (Figure 3B).

**Figure 3:**
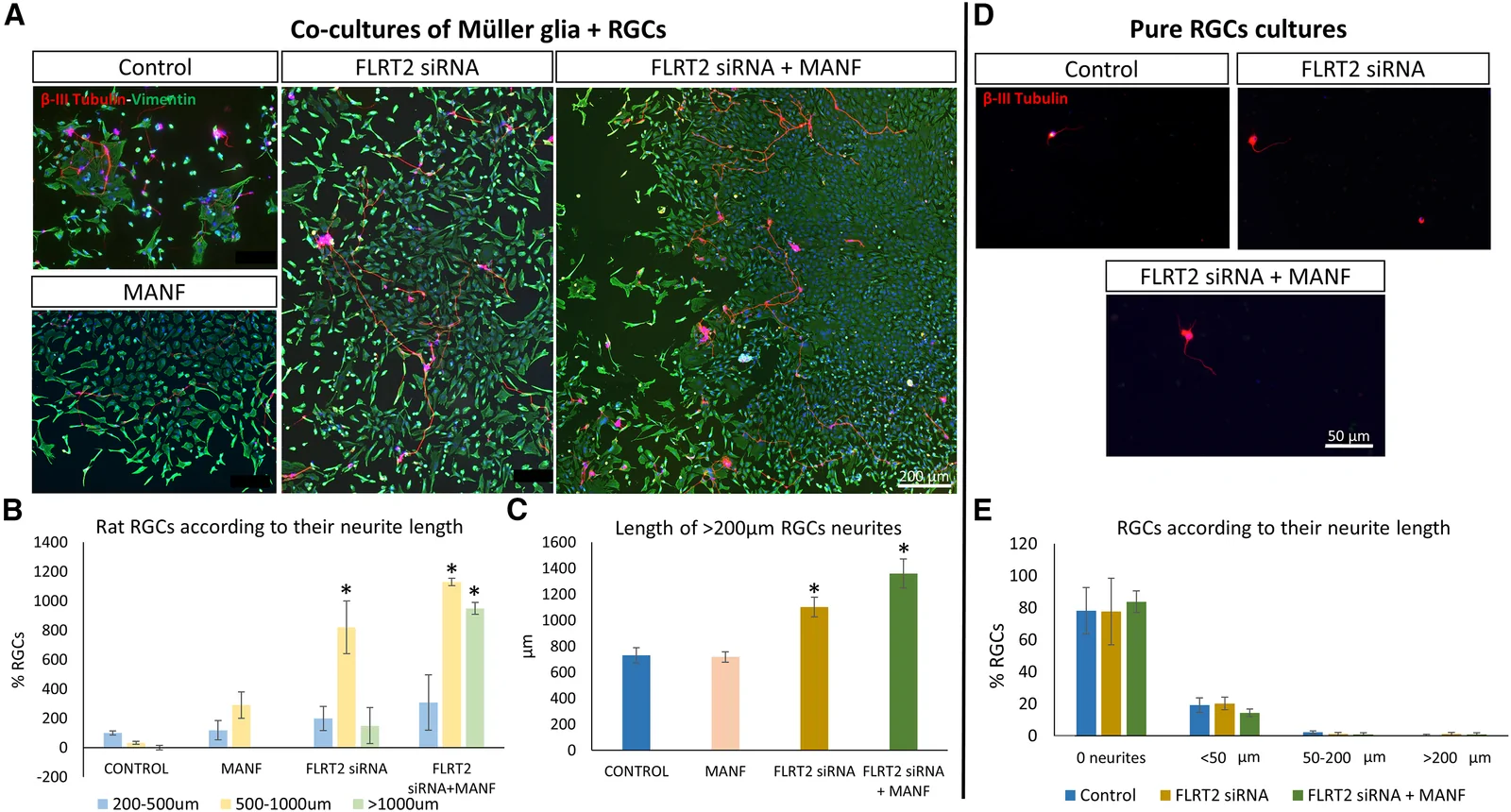
Effect of the addition of MANF and FLRT2 inhibition on neurite outgrowth in rat RGCs co-cultured with rat Müller glia and in rat highly enriched RGC cultures (A) Representative images of rat RGCs in co-culture with rat Müller glia under different conditions: control (untreated cultures), MANF treatment, FLRT2 siRNA treatment, and combined MANF and FLRT2 siRNA treatment at 6 DIV. RGCs (red) are labeled with an antibody against β-III-tubulin, while Müller glia (green) are labeled with an antibody against vimentin. Nuclei (blue) are stained with DAPI. Scale bars: 200 μm. (B) Percentage of RGCs exhibiting neurites of different lengths (200–500, 500–1,000, and >1,000 μm) in co-culture under control conditions (no treatment) and following treatment with MANF, FLRT2 siRNA, or both. (C) Quantification of rat RGC neurite length (μm) in RGCs with extended neurites (>200 μm) under control conditions (co-cultures without any treatment) and after treatment with MANF, FLRT2 siRNA, or their combination. (D) Representative images of rat RGC-enriched cultures treated with FLRT2 siRNA or a combination of MANF and FLRT2 siRNA at 6 DIV. RGCs (red) are labeled with β-III-tubulin. To confirm the purity of the cultures, Müller glia (green) are labeled with vimentin. Nuclei (blue) are stained with DAPI. Scale bars: 50 μm. (E) Classification of RGCs in highly enriched cultures based on neurite length, categorized as no neurites, short (<50 μm), medium (50–200 μm), and long (>200 μm), control (untreated cultures), following treatment with FLRT2 siRNA or a combination of MANF and FLRT2 siRNA. ∗ indicates *p* < 0.05; all statistical analyses were performed using Kruskal-Wallis nonparametric test. Data represented by mean ± SEM. Data represent 3 independent experiments (3 replicates from each).

When RGCs were categorized based on neurite length (200–500, 500–1,000, and >1,000 μm), a significant increase in the proportion of RGCs with 500–1,000 μm neurites was observed following FLRT2 siRNA treatment (820.01% ± 179.62%), with the most pronounced effect in the combined FLRT2 siRNA + MANF treatment group (1130.04% ± 24.49%) compared to the control condition (33.40% ± 10.00%). Notably, the percentage of RGCs with the longest neurites (>1,000 μm) increased dramatically rising from zero in the control condition to 950.32% ± 40.82% in the combined FLRT2 siRNA + MANF group, (Figure 3C). In contrast, treatment with MANF alone did not result in any increase in either the length of the longest neurites or the proportion of RGCs bearing neurites of any length. These findings suggest that both FLRT2 inhibition and MANF treatment synergistically promote neurite outgrowth, likely through complementary mechanisms.

To determine whether the observed effects were dependent on the presence of Müller glia, we conducted similar experiments in RGC-enriched cultures. In this instance, no significant differences in neurite outgrowth were observed following FLRT2 siRNA treatment, either alone or in combination with MANF (Figure 3E) compared to control (cultures without any treatment). This suggests that the pro-regenerative effect of FLRT2 silencing is mediated through Müller glia rather than a direct effect on RGCs. The lack of response in the absence of Müller glia supports the idea that FLRT2 inhibition modulates the Müller glia microenvironment, making it more permissive for RGC neurite extension.

Together, these results suggest that FLRT2 inhibition and MANF supplementation independently promote neurite outgrowth in rat RGCs, and their combined application exerts a synergistic effect.

## Discussion

Müller glia play a crucial role in maintaining retinal homeostasis and regulating neurite outgrowth in RGCs. In this study, we demonstrate that Müller glia derived from whales exhibit an exceptional ability to promote RGC survival and neurite outgrowth compared with Müller glial cells from other species, such as pigs and rats. This finding suggests that whale Müller glia possess unique neuroprotective and neuroregenerative properties.

It is known that Müller glia secrete trophic factors that support RGC survival and neuritogenesis.^20^^,^^21^^,^^26^^,^^27^ They express neurotrophins such as NGF, BDNF, and NT3, along with their high- and low-affinity receptors, as well as other key factors like FGFb, CNTF, and LIF.^28^^,^^29^^,^^30^^,^^31^^,^^32^^,^^33^ These molecules play crucial roles in differentiation, proliferation, neuroprotection, and RGC survival, contributing to retinal health and function.

The results from the experiments evaluating the effects of co-cultures of rat RGCs and whale Müller glia and whale Müller glia CM on rat RGCs reveal a striking enhancement in both cell survival and neurite outgrowth. The significant increase in RGC survival and the higher proportion of RGCs with extended neurites in the presence of whale Müller glia CM, compared to control conditions, suggest that soluble factors secreted by whale Müller glia play a crucial role in promoting neuronal viability and neurite growth. This is further supported by the observation that RGCs with long neurites (>200 μm) exhibited significantly increased neurite length when cultured with whale Müller glia CM, compared to control conditions. These findings imply that whale Müller glia secrete unique factors that create a highly supportive microenvironment for RGCs, potentially due to evolutionary adaptations in whales that require robust neural maintenance and repair mechanisms in their complex visual systems.

When comparing the effects of Müller glia from different species, whale Müller glia consistently outperformed rat and pig Müller glia in promoting neurite extension. The significantly longer neurites observed in RGCs co-cultured with whale Müller glia compared to rat and pig Müller glia, along with the higher percentage of RGCs with neurites exceeding 1,000 μm, suggest that whale Müller glia provide a more growth-permissive environment. This could be attributed to species-specific differences in the composition of secreted factors, such as growth factors, extracellular matrix proteins, or cytokines, which may be more potent or abundant in whale Müller glia. Additionally, the enhanced neurite outgrowth observed with Müller glia derived from both fin and sei whales further supports the notion that whale Müller glia share conserved properties among large cetaceans that promote neuronal growth. These neurotrophic characteristics may reflect evolutionary adaptations to the complex and demanding sensory environments encountered by these large mammals. Interestingly, this effect was lost when rat RGCs were cultured on immortalized whale Müller glia. Experiments on immortalized whale Müller glia showed that immortalization is likely accompanied by broader phenotypic and functional changes that may underlie their reduced ability to support extremely long neurite outgrowth. Previous work from our group has shown that immortalized Müller glia exhibit a shift toward a more dedifferentiated and reactive-like state, including increased expression of α-smooth muscle actin and reduced levels of mature glial markers such as glutamine synthetase.^34^ Such changes are consistent with cytoskeletal remodeling and altered cell-substrate interactions, which may in turn affect the mechanical properties of the glial monolayer (e.g., increased stiffness associated with a more contractile phenotype).^35^ Immortalization likely alters the secretory profile of Müller glia, shifting the balance between pro- and anti-neuritogenic factors, reducing the capacity of immortalized Müller glia to support long-distance neurite outgrowth.

To confirm the observed differences in the regenerative potential of whale and pig Müller glia, we performed a comprehensive proteomic analysis of their CM. This analysis revealed significant differences in the profile of secreted proteins, particularly those involved in neuroprotection, positive regulation of neurite outgrowth, and inhibition of neurite elongation. These contrasting secretomes underscore the unique neurosupportive properties of whale Müller glia, which likely contribute to the enhanced survival and neurite extension observed in rat RGCs. Notably, MANF—a protein known for its critical roles in neuroprotection and modulation of endoplasmic reticulum stress—was found to be markedly more abundant in whale CM compared with pig CM (mean fold change whale/pig = 12.0). Based on these findings, we selected MANF as a candidate to investigate its functional contribution to the neuroprotective and neuritogenic effects of whale Müller glia.

Furthermore, the proteomic analysis identified a set of neuroprotective proteins, which were exclusively detected in whale CM. These findings suggest that whale Müller glia possess a unique neuroprotective profile, potentially enabling them to better support RGC survival under stress conditions. Conversely, proteins associated with the negative regulation of neurite outgrowth, such as COL3A1, FRZB, SLIT3, EFNA1, and SEMA3D, were exclusively identified in pig CM. Among these, FLRT2, a known inhibitor of neurite extension, was notably absent in whale CM but highly abundant in pig CM (fold change pig/whale = 100). The absence of inhibitory factors like FLRT2 in whale CM may further explain the more permissive environment for neurite outgrowth in RGCs cultured with whale Müller glia. Together, these proteomic findings provide compelling evidence that the superior regenerative potential of whale Müller glia is driven by a combination of enriched neuroprotective and growth-promoting factors, as well as the absence of inhibitory molecules, offering valuable insights for developing therapeutic strategies targeting neurodegenerative retinal diseases.

Overall, these findings identify whale Müller glia-derived factors as a potentially valuable model for investigating neuronal survival and neurite outgrowth. The observed differences between whale and pig Müller glia may reflect species-specific characteristics of their secretory profiles that influence neuronal growth.

MANF stood out due to its significantly higher abundance in whale Müller glia CM than in pig Müller glia CM as it exerts direct neuroprotective effects in the nervous system and provides cell-protective benefits in animal models of non-neuronal diseases, including retinal damage, diabetes mellitus, liver injury, myocardial infarction, and nephrotic syndrome.^36^ Our results in co-cultures and RGC-enriched cultures indicate that MANF, selected from the proteomic analysis of whale Müller glia secretions, did not independently promote RGC neurite outgrowth under the conditions tested but enhanced this response when combined with FLRT2 silencing. Several studies have shown that MANF is upregulated in RGCs and adjacent glial cells under stress conditions,^37^ promoting cell viability and reducing apoptosis in response to hypoxia and endoplasmic reticulum stress. Additionally, under oxidative stress induced by H_2_O_2_, MANF secreted by neural and glial tissues prevented a decrease in neurite outgrowth while maintaining neural marker expression.^38^ MANF primarily enhances intracellular pro-growth signaling pathways, including Akt/mTOR and Erk/mTOR,^39^ which promote protein synthesis and cytoskeletal dynamics required for neurite extension. However, these intracellular growth programs may require a permissive extracellular environment to overcome inhibitory guidance cues, suggesting that FLRT2 inhibition relieves repulsive signaling and thereby enables MANF-dependent neurite outgrowth. Together, these findings support the idea that MANF’s neuroprotective and regenerative effects may be context-dependent, requiring specific stress conditions to exert its full potential.

Furthermore, the downregulation of FLRT2, a protein implicated in the negative regulation of neurite outgrowth, significantly enhanced RGC neurite extension in co-cultures. Notably, our study is the first to directly inhibit FLRT2 in RGCs using a siRNA approach. Previous studies had not directly tested FLRT2 inhibition but instead inferred its role through *ex vivo* stripe assays, where ectopic FLRT2 expression was introduced. In these assays, 18% of neurons—comprising a mixed population that included starburst amacrine cells and direction-selective ganglion cells—were repelled by FLRT2 stripes. Additionally, neurons transfected to overexpress FLRT2 actively avoided Unc5C protein stripes, suggesting this repulsive guidance response.^40^ Our findings build upon this previous evidence, demonstrating that targeted FLRT2 inhibition can enhance RGC neurite outgrowth, highlighting its potential as a therapeutic target for promoting retinal regeneration. Moreover, the lower representation of proteins associated with inhibition of neurite growth in whale Müller glia CM, compared with pig CM, suggests that differences in inhibitory signals may contribute to the observed effects. This observation supports further investigation of growth-inhibitory mechanisms as modulators of neurite outgrowth and neuronal repair.

Additionally, we found that FLRT2 inhibition did not affect neurite outgrowth in RGC-enriched cultures, suggesting that its effects are mediated indirectly through Müller glia rather than acting directly on RGCs. This highlights the crucial role of glial cells in shaping the regenerative microenvironment and suggests that targeting FLRT2 within Müller glia may be a promising strategy to enhance neuronal repair. Interestingly, our findings suggest that FLRT2 inhibition exerts its effects through Müller glia rather than directly on RGCs, highlighting the importance of the glial microenvironment in regulating neurite outgrowth. At present, however, the precise cellular mechanisms underlying FLRT2 action remain unresolved. Given its nature as a membrane-associated adhesion and signaling molecule, it may act either by modulating Müller glial surface interactions with RGCs or by influencing glial state and associated secretory and extracellular properties. Further glia-specific manipulation of FLRT2 will therefore be required to dissect its exact site of action and downstream pathways.

Several studies have demonstrated that suppressing intrinsic inhibitory pathways can significantly enhance RGC neurite regeneration.^41^ For instance, knockout of PTEN,^7^^,^^10^ deletion of KLF9,^42^ or more recently, depletion or inhibition of core stress granule proteins such as G3BP1^43^ have all been shown to promote robust neurite elongation in RGCs. Interestingly, in the context of whale Müller glia, it is plausible that naturally low levels of these intrinsic inhibitory factors, or the active secretion of modulatory factors by Müller glia, may underlie the remarkable RGC elongation observed. These findings suggest that whale Müller glia might exert a unique, naturally permissive influence on neurite growth that parallels or even surpasses the effects achieved through genetic or pharmacological inhibition of well-established intrinsic suppressors.

These results underscore the unique regenerative potential of whale Müller glia and provide novel insights into glia-mediated mechanisms of neuroprotection and axonal repair, which could have significant implications for developing therapeutic strategies for retinal neurodegenerative diseases. The exceptional ability of whale Müller glia to promote RGC survival and robust neurite outgrowth suggests that their secreted factors create an exceptionally supportive microenvironment, likely shaped by evolutionary adaptations to the demanding sensory environments of large marine mammals. Importantly, proteomic analysis revealed that pig Müller glia CM was enriched in proteins associated with axon guidance, adhesion, and neurite growth inhibition, whereas several of these inhibitory-associated factors were reduced or absent in whale CM. Moreover, functional inhibition of FLRT2 partially rescued neurite outgrowth, supporting its role as a negative regulator in this context. Our findings highlight the balance between neurotrophic support and inhibitory signaling in the regulation of neuronal survival and neurite outgrowth, suggesting that reduced inhibitory signaling may contribute alongside the presence of trophic factors.

By identifying key molecules such as MANF and FLRT2 as modulators of neurite outgrowth, our study lays the groundwork for future research aimed at harnessing glial-derived factors to enhance retinal repair. Notably, the most pronounced effects of whale Müller glia CM were observed in RGCs extending the longest neurites, suggesting that specific RGC subtypes may be particularly responsive to whale-derived trophic signals. Previous studies have also reported subtype-dependent differences in neurite outgrowth and regenerative capacity in response to various treatments,^41^^,^^44^^,^^45^^,^^46^^,^^47^^,^^48^ supporting the idea that these differential responses could be leveraged therapeutically. Recently, Lukomska et al*.*^3^ proposed that the regenerative capacity of specific RGC subtypes may depend on the availability of subtype-permissive extracellular substrates within the injured optic nerve microenvironment. In particular, they have reported that fibronectin supports axonal growth of selected adult RGC subtypes, potentially through differential expression of fibronectin-binding integrins. In agreement with this concept, our group has previously demonstrated that adult RGCs preferentially extend neurites on laminin-containing substrates compared to other extracellular matrix conditions^49^ and that distinct RGC subtypes display differential resistance responses following injury in a chronic model of glaucoma.^50^ Nonetheless, although differential fibronectin production by whale Müller glia cannot be excluded,^35^^,^^51^ such differences in extracellular matrix deposition are not necessarily reflected in CM-based analyses. Consistent with this, CM from whale Müller glia promoted neurite elongation even in the absence of direct cell-cell or cell-substrate interactions, supporting a major contribution of soluble secreted factors to the observed effects. In this context, the species-dependent differences identified in the Müller glia secretome may contribute to the generation of variable permissiveness for specific RGC populations to the extracellular environment. Thus, whale Müller glia may provide a supportive secretory environment capable of enhancing neurite extension in specific RGC subpopulations within the enriched cultures.

Importantly, the unique opportunity to study Müller glia from the largest mammals on Earth has enabled us to uncover evolutionarily refined mechanisms of neuronal survival and neurite elongation. These discoveries highlight the possibility that certain molecules and pathways involved in neurite extension are highly conserved and universally recognized by neurons across distantly related species, offering a promising perspective for developing regenerative therapies applicable across a broad range of patients.

Although this study reveals the exceptional ability of whale Müller glia to promote RGC survival and neurite outgrowth, several limitations constrain the interpretation and translational potential of these findings. First, the rarity and ethical constraints surrounding the acquisition of whale tissue limit experimental replicates and broader validation across individual variability or additional cetacean species even when the authors could study 2 whales in optimal conditions. Second, while proteomic analyses identified different candidate modulators, the functional validation focused on MANF and FLRT2, and potential synergistic or context-dependent effects of other secreted factors remain unexplored. Furthermore, the molecular mechanisms underlying the glia-mediated, indirect regulation of neurite outgrowth require deeper investigation. To advance these findings toward therapeutic application, future studies should include *in vivo* validation in disease models, characterization of human Müller glial analogs, and exploration of delivery strategies for key trophic factors.

Future investigation of the molecular composition of whale Müller glia secretomes and their interactions within retinal neuronal and glial networks may provide further insight into the mechanisms regulating neuronal survival and neurite outgrowth. These findings provide a basis for future studies examining whether such mechanisms could inform neuronal repair strategies for glaucoma and other optic neuropathies.

### Limitations of the study

The availability of primary Müller glia from large baleen whales inherently limits the number of biological samples that can be obtained for comparative studies. In addition, the cross-species experimental design used here was intended to identify species-associated differences in Müller glial support and candidate molecular signals rather than to establish the contribution of individual factors *in vivo*. Further studies using complementary experimental models will help determine how the identified candidates contribute to Müller glia-mediated neuronal support in the mammalian retina.

## Resource availability

### Lead contact

Requests for further information, resources, and reagents should be directed to and will be fulfilled by the lead contact, Xandra Pereiro (xandra.pereiro@ehu.eus).

### Materials availability

This study did not generate new, unique reagents.

### Data and code availability

• The mass spectrometry proteomics data have been submitted to the ProteomeXchange Consortium via the PRIDE partner repository (accession number: PXD082515; https://www.ebi.ac.uk/pride/archive/projects/PXD082515).
• This paper does not report original code.
• Any additional information required to reanalyze the data reported in this paper is available from the lead contact upon request.

## Acknowledgments

This study was supported by Grupos Consolidados 10.13039/501100003086Gobierno Vasco (IT1886-26) and MINECO-Retos MICIU/10.13039/501100011033AEI/10.13039/501100011033 (PID2023-152778OB-I00). We thank the organizations that assisted us in obtaining the whale eyes: AMBAR in the Basque Country, CEPESMA in Asturias, and Oceanogràfic Valencia.

## Author contributions

Conceptualization, X.P. and E.V.; methodology, X.P., N.R., M.B., S.M.H., F.E., and M.A.; investigation, X.P. and E.V.; visualization, X.P. and L.P.-L.; supervision, X.P., S.M.H., and E.V.; writing – original draft, X.P.; writing – review and editing, X.P., N.R., L.P.-L., S.M.H., and E.V.

## Declaration of interests

The authors declare no competing interests.

## STAR★Methods

### Key resources table

**Table:** 

REAGENT or RESOURCE	SOURCE	IDENTIFIER
**Antibodies**		

Mouse anti-β-III-Tubulin (1:1,000)	Abcam	Cat# ab7751; RRID: AB_306045
Rabbit anti-CRALBP (1:1,000)	Abcam	Cat# ab154898
Mouse anti-GFAP (1:1,000)	Sigma-Aldrich	Cat# G3893; RRID: AB_477010
Rabbit anti-Glutamine synthetase (GS) (1:1,000)	Abcam	Cat# ab49873; RRID: AB_880241
Rabbit anti-P75 (1:2,000)	Abcam	Cat# ab8877; RRID: AB_306830
Rabbit anti-Vimentin (1:1,000)	Abcam	Cat# ab92547; RRID: AB_10562134

**Chemicals, peptides, and recombinant proteins**		

Papain	Worthington Biochemical	Cat# LK003176
DNase	Worthington Biochemical	Cat# LK003170
B-27 Supplement (50*X*)	Gibco	Cat# 17504044
Lipofectamine RNAiMAX Transfection Reagent	Thermo Fisher Scientific	Cat# 13778150
Opti-MEM I Reduced Serum Medium	Gibco/Thermo Fisher Scientific	Cat# 31985062

**Critical commercial assays**		

Papain Dissociation System	Worthington Biochemical	Cat# LK003150

**Deposited Data**		

Mass spectrometry proteomics dataset	This study (ProteomeXchange Consortium- PRIDE; https://www.ebi.ac.uk/pride/archive/projects/PXD082515)	PXD082515

Experimental models Cell lines		

Immortalized sei whale Müller glia (W21M)	Pereiro et al.^34^^,^^52^	W21M

**Software and algorithms**		

Proteome Discoverer	Thermo Fisher Scientific	v2.4.1.15
IBM SPSS Statistics	IBM	v24

### Experimental model and study participant details

#### Animals and tissue sources

This study was carried out in strict accordance with the recommendations in the Guide for the Care and Use of Laboratory Animals. The experimental protocol met European (2010/63/UE) and Spanish (RD53/2013) regulations for the protection of experimental animals, and it was approved by the Ethical Committee for Animal Welfare of the University of the Basque Country. All animal experimentation adhered to the ARVO Statement for the Use of Animals in Ophthalmic and Vision Research.

Eyes (∼1 kg) from adult beached Balaenoptera physalus (fin whale) and Balaenoptera borealis (sei whale) whales (*n* = 2) were collected 24 h post mortem. The exact age of the animals was unknown. Once the eyes were removed from the animals, they were maintained at 4°C until they were taken to the laboratory.

Porcine eyes were obtained from approximately 1-year-old male and female pigs at a local slaughterhouse and transported to the laboratory in cold CO2-independent medium (Life Technologies, Carlsbad, CA, USA) containing 0.1% gentamicin (Life Technologies, Carlsbad, CA, USA).

Rat eyes were obtained from 2-month-old female Sprague Dawley rats (200–250 g). Animals were housed on a 12-h light-dark cycle with *ad libitum* access to food and water, and they were sacrificed humanely by exposure to CO2.

### Method details

#### Whale, pig and rat müller glia culture establishment

Retina isolation and Müller glia culture were established following Pereiro et al., 2020.^53^ Briefly, the retinas were dissected out and fragments of 8 mm of diameter of the whale and porcine retinas, or the entire rat retina, were digested with Sterile Earle`s Balanced Sat Solution (EBSS) with papain (20 U/mL) and DNase (2000 U/mL; Worthington) during 30 min at 37°C. Enzymatic digestion was stopped by Dulbecco`s Modified Eagle`s Medium (DMEM; Gibco-Life Technologies) containing 10% Fetal Bovine Serum (FBS) and the retina was mechanically dissociated. Cell homogenate was centrifuged at 1200 rpm 5 min and the pellet was resuspended in DMEM+10%FBS. Approximately, 10^5^ cells were seeded in 6 well plates coated with poly-L-Lysine (100 μg/ml; Sigma-Aldrich) and laminin (10 mg/ml: Sigma-Aldrich). Whale cultures were maintained in a humidified incubator at 37°C in an atmosphere of 5% CO_2_. The medium was totally changed on 1 day of culture and half volume of the medium was replaced every 2 days until the cultures were confluent. Most experiments were performed using whale Müller glial cells maintained in primary culture without any subculturing. However, cells were also expanded to early (passage 4) and advanced (passage 7) passages to evaluate the potential impact of cellular aging on their neurotrophic capacity.

#### Culture and immortalization of whale müller glia

Primary whale Müller glial cells were isolated from post-mortem eye tissue of Sei Whale as previously described.^34^ Primary Müller glia were immortalized (W21M) with the simian virus 40 (SV40) large T-antigen commonly used to immortalize human cell lines, as detailed in Pereiro et al., 2022,^34^ Pereiro and Vecino.^52^ Cells were maintained at 37°C in a humidified atmosphere with 5% CO_2_ and subcultured upon reaching ∼80% confluency.

#### Whale müller glia conditioned media collection

Conditioned media (CM) was collected when whale Müller glia cultures had reached confluence (day 15), first washing the wells three times with DMEM medium supplemented with 1% l-glutamine and 0.1% gentamicin (Thermo-Fisher Waltham, MA, USA). DMEM was then added to each well and left for 3 h before the medium was changed to eliminate the rest of the FBS. Fresh DMEM was then added for 2 days before it was collected and sterilized by passing through a 0.22 μm filter. The CM was frozen in aliquots at −20 °C. The same procedure was applied to obtain pig and rat CM.

#### Highly enriched rat RGC cultures

Retinal ganglion cell cultures were prepared as described previously.^20^^,^^26^^,^^27^^,^^33^^,^^49^^,^^54^^,^^55^^,^^56^^,^^57^^,^^58^^,^^59^ Briefly, the retinas from rats were dissected out to obtain a mixed suspension of retinal cells. Retinal tissue was dissociated enzymatically using the Papain Dissociation Kit (Worthington Biochemical, Lakewood, NJ, USA) according to the manufacturer's instructions. Briefly, the tissue was digested for 90 min at 37 °C in 0.2% activated papain with 10% DNAse I and the tissue was then disaggregated by gentle trituration using pipette tips of decreasing diameter. RGCs were purified by an ovalbumin gradient-based cell separation step. After purification, RGCs were plated on 13 mm poly-L-lysine (100 μg/mL, Sigma-Aldrich, St. Louis, MO, USA) and laminin (10 μg/mL: Sigma-Aldrich, St. Louis, MO, USA) coated glass coverslips in 24-well plates. The rat cells were then seeded at 10^5^ viable cells per well. The cultures were maintained in Neurobasal A medium (Gibco-Life Technologies, Carlsbad, AC, USA) supplemented with 2% B27, 1% L-glutamine (2 mm: Life Technologies, Carlsbad, CA, USA) and 0.1% gentamicin (50 mg/mL: Life Technologies, Carlsbad, CA, USA). Although the culture medium itself was not used as a purification step for RGCs, serum-free culture conditions were applied to prevent proliferation of residual Müller glial cells. Rat RGCs were seeded on 80% confluent whale, pig and rat Müller glia cultures.

To evaluate the effect of conditioned media (CM) obtained from Müller glia cultures of whale, pig and rat origin, RGCs were cultured either in standard NBA/B27 medium (control) or in a 1:1 mixture of NBA/B27 medium and the respective CM. All the media used contained 1% L-glutamine and 0.1% gentamicin. Cultures were kept at 37 °C in a 5% CO_2_ humidified incubator for 6 days, and the medium was changed every 3 days. At the end of the culture period (day 6), cells were fixed with methanol at −20 °C for 10 minutes.

At least three replicates were performed for each analyses described, repeating each independent experiment a minimum of three times.

#### Immunocytochemistry

RGC-enriched cultures and co-cultures were analysed by immunocytochemistry. Whale Müller glia were further characterized by immunostaining for Müller glia-specific markers (Figure S2). Cells were washed in Phosphate Buffered Saline (PBS, pH 7,4), fixed in methanol at -20°C for 10 min and non-specific antigen binding was blocked with blocking buffer (0.1% triton X-100 and 3% Bovine Serum Albumin BSA in PBS). The primary antibodies were diluted in blocking buffer and incubated overnight at 4°C. The cells were washed three times in PBS and cultures were incubated 1 h at room temperature with the corresponding secondary antibodies at a dilution of 1:1,000; Alexa Fluor 488 and Alexa Fluor 555 conjugated goat anti-mouse and goat anti-rabbit antibodies (Invitrogen). After three washes, the coverslips were mounted with Fluor-save Reagent (Calbiochem).

Müller cells were observed with an epifluorescence microscope (Zeiss) coupled to a digital camera (Zeiss Axiocam MRM, Zeiss).

### Quantification and statistical analysis

RGCs were analysed on an epifluorescence microscope (Zeiss, Jena, Germany) coupled to a digital camera (Zeiss Axiocam MRM, Zeiss, Jena, Germany). The images were taken under the same conditions of intensity and exposure time. For conditions displaying extensive neurite outgrowth, images were acquired as mosaics to allow visualization of the full length of RGC neurites. Importantly, all individual tiles were acquired using identical magnification and imaging settings across all experimental conditions, ensuring comparability at the acquisition level.

At least three coverslips were fully analysed for each experimental condition and from a minimum of three independent experiments. In RGC cultures, all βIII-tubulin-positive cells present in each coverslip (132.7 mm^2^) were counted to determine RGC density and the total number of RGCs surviving in each condition was recorded. Under control conditions (RGC-enriched cultures without Müller glia or CM from Müller glia), an average of approximately 100 RGCs per coverslip (0.75 cells/mm^2^) was counted. Accordingly, a total of roughly 900 cells was set as 100% and survival in all other conditions was expressed relative to this value for the statistical analyses depicted in the graphs.

In addition, the RGC neurites length was quantified from the images using ZEN software (Zeiss, Jena, Germany) by manually tracing the longest neurite of each cell, yielding the measurement of its maximal extension and were classified as: 1) cells with no neurites; 2) cells with the longest neurite <50 μm; 3) cells with the longest neurite between 50 and 200 μm; 4) cells with the longest neurite between 200 and 1000 μm; and 5) cells with neurites longer than 1000 μm. In addition, the largest neurites, longer than 200 μm, were measured and classified as 1) cells with the longest neurite between 200 and 500 μm; 2) cells with the longest neurite between 500 and 1000 μm and 3) cells with neurites longer than 1000 μm.

The means and standard error of mean (SEM) are presented for each condition. Statistical analyses were carried out using IBM SPSS Statistical software v.24-0. The data from the different experimental conditions were compared using the non-parametric Mann-Whitney U test analysis. When we compare more than two independent groups, Kruskal-Wallis nonparametric test were performed and if the Kruskal–Wallis test was significant, a post-hoc analysis, Dunn test, was performed on order to determine which groups differ from each other group. Differences were considered significant for all tests at *p*-value ≤ 0.05.

#### Sample preparation for mass spectrometry

Each CM was prepared in technical triplicates using 350μl each for proteolysis by a modified filter-aided sample preparation (FASP) digest protocol.^60^ Briefly, after reducing all proteins with DTT for 30min at 60°C, samples were diluted with 8M Urea to a final concentration of >4M urea, carbamidomethylated by addition of iodacetamide and then centrifuged on the filter, washed three times with 8M urea and twice with 50 mM ammoniumbicarbonate (ABC). Proteins were digested in ABC with Lys-C and trypsin overnight and resulting peptides collected by centrifugation. Samples were acidified with 0.5% trifluoroacetic acid (TFA) prior to mass spectrometric analysis.

#### Mass spectrometry of CMs

LC-MSMS analysis was performed on a Q Exactive HF mass spectrometer (ThermoFisher Scientific, Waltham, Massachusetts, USA) online coupled to an Ultimate 3000 RSLC nano-HPLC (Dionex, Sunnyvale, California, USA). CMs from whale and pig Müller glia were automatically injected and loaded onto the C18 trap column and after 5 min eluted and separated on the RP-C18 analytical column (μPCA, 2m length; PharmaFluidics, Gent, Belgium) by a 240 min non-linear acetonitrile gradient (from 3 % to 43% acetonitril) at a flow rate of 300nl/min. MS spectra were recorded at a resolution of 60,000 and after each MS1 cycle, the 10 most abundant peptide ions were selected for fragmentation. Dynamic exclusion was applied for a 50 second duration.

#### Analysis and quantification of mass spectrometry data

The resulting RAW files were analysed in Thermo Proteome Discoverer (PD) 2.4.1.15. Database searches were performed with SEQUEST HT (as a node in PD) with the following settings: trypsin digestion, 1 maximum missed cleavage, precursor mass tolerance of 10 ppm, fragment mass tolerance of 0.2 Da, a fixed modification of +57.021 Da (carbamidomethylation) on cysteine, and variable modifications of +15.995 Da (oxidation) on methionine and N-terminal Met-loss+Acetyl - 89.030 Da. A reverse database search was performed using SEQUEST HT to determine the spectral false discovery rate (FDR), and peptide-spectrum matches (PSM) were filtered to <1 % False FDR based on Percolator q-value, search engine rank 1 and posterior error probability <0.01.

The FASTA databases used were either the SwissProt human database (release February 2017; 20,237 Sequences), the Ensembl pig database (Release 75, Sscrofa10.2; 25,859 Sequences) or a database generated from the NCBI *Balaenoptera_physalus* (release November 2019; 19,991 Sequences). An area-based label free protein quantification was performed, and proteins with an abundance fold/ratio >1.5 in favour of the whale sample in any of the searches were considered for further analyses.

Prior to quantitation, the Spectrum Files RC node in PD was utilized to perform spectrum recalibration. In addition, the MS1 intensity quantitative tool Minora Feature Detector was utilized for label-free quantitation for area under the curve calculations for all unique plus razor peptides detected per unique protein satisfying strict parsimony principle. Normalization was performed using the total peptide amount to account for random errors.

The proteins listed were ordered according to the fold change obtained, and the proteins selected, out of a total of 1223 proteins, were categorized based on their functions attributed in the UniProt database.

#### Treatment of cultures with MANF and FLRT2 siRNA

On the third day of co-cultures of rat Müller glia and RGCs and enriched rat RGCs, Mesencephalic Astrocyte-Derived Neurotrophic Factor (MANF) protein (100ng/ml, PeproTech), as well as small interfering RNA [Fibronectin Leucine-Rich Transmembrane protein 2 (FLRT2) siRNA (20 nM)] (following the manufacturer's protocol, Sigma-Aldrich), were added individually or in combination to the corresponding wells of a 24-well plate. Protein solutions were prepared in culture medium at the desired concentrations. For FLRT2 silencing, siRNA-lipoplexes were prepared by first diluting Lipofectamine RNAiMAX (Thermo Fisher) to a final concentration of 0.2% v/v in Opti-MEM medium. Separately, siRNA was diluted to a final concentration of 20 nM in Opti-MEM. Both solutions were then combined, gently mixed, and incubated at room temperature for 5 minutes before being added to the cultures.

After the addition of proteins and siRNA, cultures were incubated for three days to allow interaction with the cells. Subsequently, cultures were fixed and the neurites of RGCs length analysed as previously described. At least three independent experiments were performed, each including a minimum of four replicates per condition to ensure reproducibility and statistical robustness.
