# Supplementary material for: Whale Müller glia create a permissive environment supporting retinal ganglion cell survival and neurite outgrowth
Source: iScience. 2026 Sep 25;29(10):117637. doi: 10.1016/j.isci.2026.117637 (PMC13635513; doi:10.1016/j.isci.2026.117637)
Supplement: Supplementary file 1 [file mmc1.pdf]

iScience, Volume *29*

Supplemental information

Whale Müller glia create a permissive environment supporting retinal ganglion cell survival and neurite outgrowth

Xandra Pereiro, Noelia Ruzafa, Laura Prieto-López, Marcel Blindert, Stefanie M. Hauck, Félix Elortza, Mikel Azkargorta, and Elena Vecino

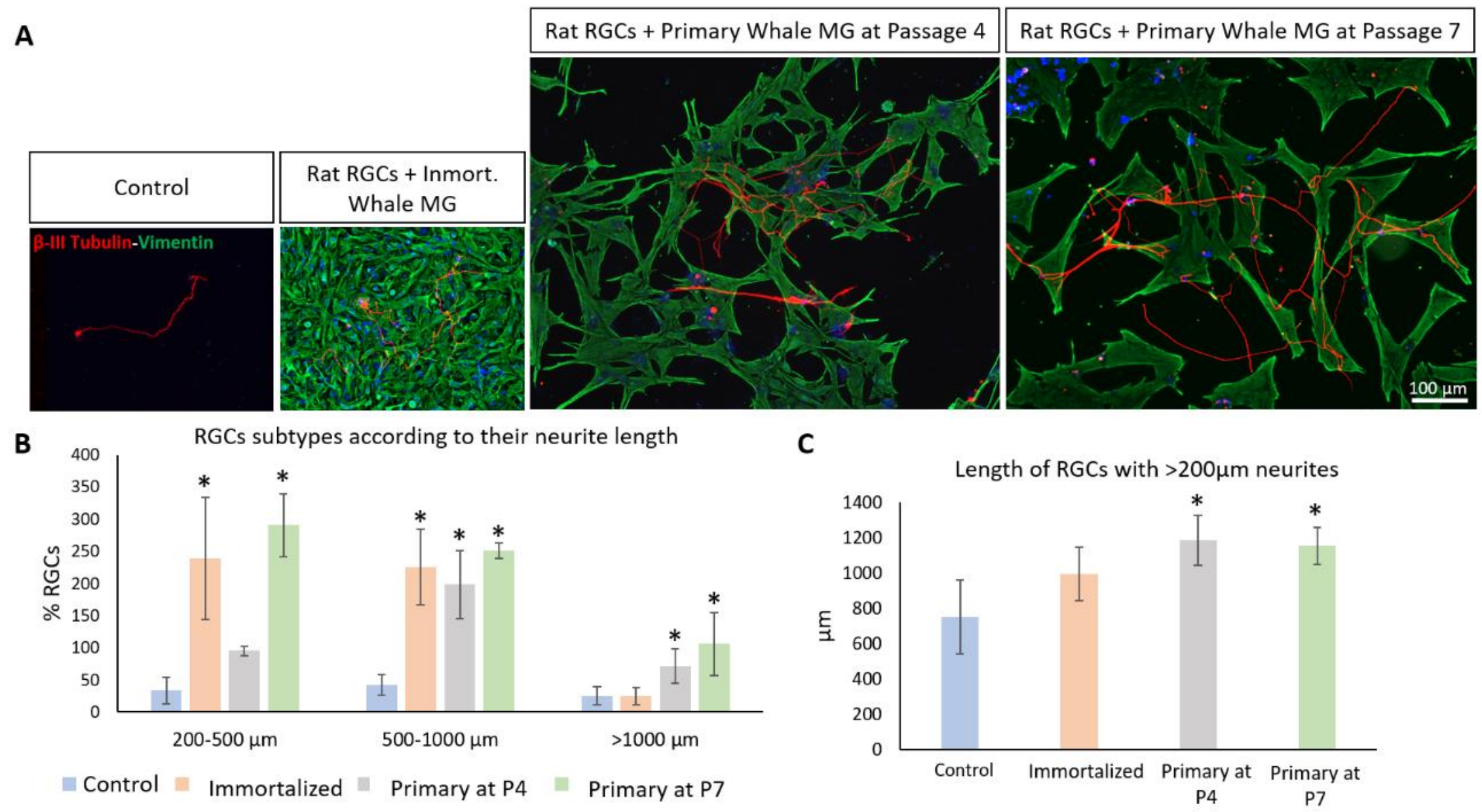

Fig. S1. Comparative analysis of neurite outgrowth in rat RGCs co-cultured with immortalized and primary whale Müller glia at passage 4 and 7. (A) Images of rat RGCs in control (RGC-enriched cultures) and in co-culture with immortalized (W21M) and primary whale Müller glia at passages 4 and 7. RGCs (red) labelled with an antibody against β-III-Tubulin and Müller glia (green) labelled with an antibody against Vimentin, nuclei (blue) stained with DAPI. Scale bar: 100µm. **(B)** Percentage of rat RGCs with 200-500µm, 500-1000µm and >1000µm neurites in culture with immortalized and primary whale Müller glia at passages 4 and 7. Primary Müller glia, particularly at higher passages, significantly enhance neurite outgrowth in rat RGCs compared to control. **(C)** Analysis of the length of >200µm rat RGCs neurites in co-culture with immortalized and primary whale Müller glia at passages 4 and 7. Primary whale Müller glia increase significantly the length of RGCs with >200µm neurites. * indicates $p<0.05$; all statistical analyses were performed using Kruskal-Wallis nonparametric test. Data represented by Mean ± SEM. Data represent 3–4 independent experiments (4 replicates from each).

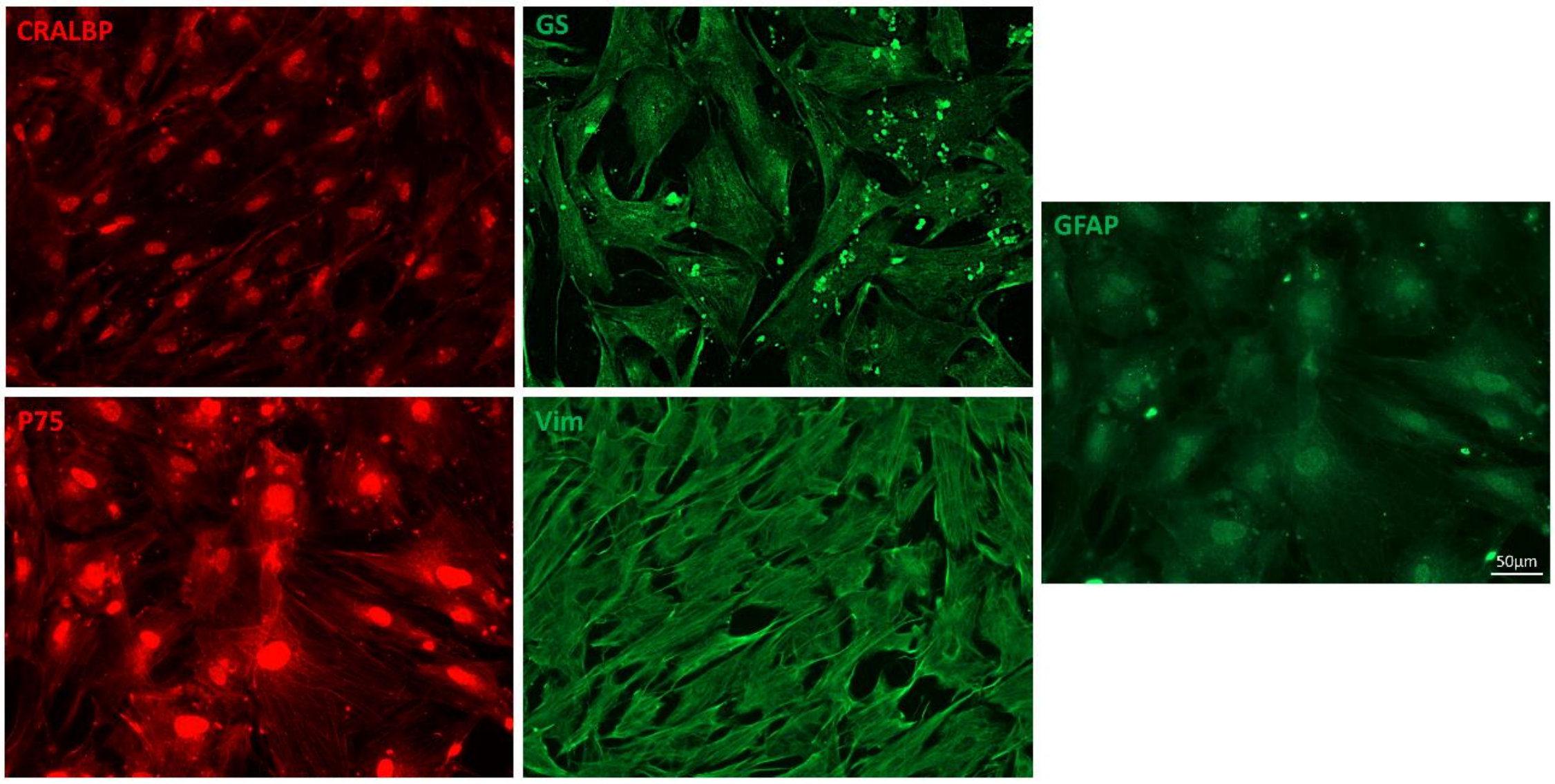

Fig. S2. Characterization of whale Müller glia by expression of typical Müller cell markers. Representative immunofluorescence images of whale Müller glia showing the expression of characteristic Müller glial markers. Cells were labelled with antibodies against CRALBP and p75 (red), and against glutamine synthetase (GS), Vimentin and GFAP (green). The expression pattern confirms the preservation of typical Müller glia molecular markers in cultured whale Müller cells. Scale bar: 50 µm.
